# Serum calcitonin gene-related peptide in patients with persistent post-concussion symptoms, including headache: a cohort study

**DOI:** 10.1007/s00415-024-12181-y

**Published:** 2024-01-17

**Authors:** Peter Preben Eggertsen, Johan Palmfeldt, Henrik Winther Schytz, Debbie Hay, Rikke Katrine Jentoft Olsen, Jørgen Feldbæk Nielsen

**Affiliations:** 1 Department of Clinical Medicine, Hammel Neurorehabilitation Centre and University Research Clinic, Aarhus University, Voldbyvej 15A, 8450 Hammel, Denmark; 2 Department of Clinical Medicine, Research Unit for Molecular Medicine, Aarhus University Hospital and Aarhus University, Palle Juul-Jensens Boulevard 99, 8200 Aarhus N, Denmark; 3 Faculty of Health and Medical Sciences, Department of Neurology, Danish Headache Center, Rigshospitalet Glostrup, University of Copenhagen, Valdemar Hansens Vej 5, 2600 Glostrup, Denmark; 4 Department of Pharmacology and Toxicology, University of Otago, 362 Leith Street, Dunedin North, Dunedin, 9016 New Zealand

**Keywords:** CGRP, Concussion, Mild traumatic brain injury, Biomarker, PCS, Post-traumatic headache

## Abstract

**Background:**

Calcitonin gene-related peptide (CGRP) plays an important role in migraine pathophysiology, and post-traumatic headache (PTH) frequently presents with migraine-like features. Despite several clinical similarities, few studies have explored CGRP in PTH and concussion. This study investigates serum CGRP levels in patients with persistent post-concussion symptoms (PPCS), including PTH.

**Methods:**

This cohort study was based on serum samples from individuals aged 18–30 years with PPCS who participated in a previously published randomized controlled trial of a non-pharmacological intervention. The primary outcome was serum CGRP concentrations, determined at baseline before randomization and at follow-up 7 months later, using an enzyme-linked immunosorbent assay (ELISA). CGRP levels at baseline were compared with healthy anonymous blood donors in the same age group.

**Results:**

Baseline serum samples were collected from 86 participants with PPCS. The participants were most often female (78%) and migraine-like headache was the most frequent headache phenotype (74%). Serum CGRP levels were higher in participants with PPCS than in 120 healthy individuals (median: 158.5 pg/mL vs. 76.3 pg/mL, p = 0.050). A stratified analysis revealed that females with PPCS had a fivefold higher median than healthy females (166.3 pg/mL vs. 32.1 pg/mL, p = 0.0006), while no differences were observed in males (p = 0.83). At follow-up, CGRP levels decreased with a median change of  – 1.3 pg/mL (95% confidence interval:  – 17.6–0, p = 0.024).

**Discussion:**

Elevated serum levels of CGRP in patients with PPCS and a decrease over time suggest an involvement of CGRP in PTH/PPCS. If confirmed in other studies, it could pave the way for CGRP-targeted therapies, which could have clinical significance.

**Supplementary Information:**

The online version contains supplementary material available at 10.1007/s00415-024-12181-y.

## Introduction

Concussion is a prevalent injury accounting for up to 98% of all traumatic brain injuries (TBI) [1], and is often referred to as mild TBI [2]. In Europe, the incidence rate of TBI is approximately 300 cases per 100,000 person-years [3], whereas in New Zealand it may be even higher with 790 cases per 100,000 person-years [4]. These incidence rates result in an estimated 55.9 million concussions occurring globally each year [5]. While the majority of those affected recover, up to 30% develop persistent post-concussion symptoms (PPCS) [6] with post-traumatic headache (PTH) being one of the most frequent symptoms [7]. Treating post-traumatic headache (PTH) is a challenge due to the lack of an evidence-based treatment strategy [8], and despite its high prevalence, the underlying pathophysiology remains unknown. Current proposed management guidelines are based on the headache phenotype of PTH [9], which is often migraine-like [10].

During the last 30 years, calcitonin gene-related peptide (CGRP) has substantially increased the understanding of migraine pathophysiology. Initial research revealed increased plasma CGRP levels in migraine patients during attacks [11], and later, its headache-inducing properties were discovered [12]. The importance of CGRP is underlined by CGRP antibodies being used as preventive medication in episodic and chronic migraine. Although the precise pathophysiological mechanism of CGRP remains to be elucidated, it may contribute to migraine due to its ability to cause both peripheral and central sensitization within the trigeminovascular system [13].

Despite the clinical similarities between PTH and migraine, remarkably few studies have examined CGRP concentrations in individuals with concussion who develop PPCS/PTH. One of the few studies available in this field found lower blood CGRP levels in PTH, contrasting with the findings of migraine studies [14]. Due to the paucity of evidence, we aimed to replicate this finding by assessing serum CGRP levels in patients with PPCS/PTH 2–6 months following a concussion.

## Methods

### Design

This cohort study was based on serum samples from participants included in a recently published open-label parallel-group randomized controlled trial (RCT) conducted at Hammel Neurorehabilitation Centre and University Research Clinic and The Research Clinic for Functional Disorders and Psychosomatics, Aarhus University Hospital in Denmark. The RCT study showed that a new interdisciplinary intervention, Get going After concussIoN (GAIN), significantly lowered PPCS symptoms compared with enhanced usual care (EUC) [15]. The 8-week interdisciplinary intervention was non-pharmacological, based on cognitive behavioral therapy and gradual return to activities, and included both group sessions and individual sessions with a physio/occupational therapist and a neuropsychologist [15]. As outlined in the preregistered analysis plan (referenced in the ‘Ethics Approval and Registrations’ section), we did not anticipate any influence of the non-pharmacological intervention on CGRP levels within the cohort. Consequently, both intervention arms were combined at follow-up akin to a cohort study for the primary outcome.

### Participants and inclusion/exclusion criteria

Participants were recruited between March 2015 to September 2017 from general practitioners and hospitals in the Central Denmark Region. Eligible patients were 18–30 years old and had experienced PPCS for 2–6 months. Concussion was defined according to the recommendations from the World Health Organization (WHO) Task Force [16]. Additionally, a direct head trauma was required to rule out pure acceleration-deceleration traumas. PPCS were defined as having a Rivermead Post-Concussion Questionnaire (RPQ) [17] score ≥ 20. Patients with severe brain injury, previous concussion with symptoms > 3 months, drug/alcohol abuse, severe somatic or psychiatric conditions that impeded participation in the intervention, and patients who could not speak Danish were excluded. The inclusion/exclusion criteria were thus identical to those of the previously published RCT [15], except that patients below 18 years of age were not included in this study.

### Assessment for eligibility

Potential PPCS patients referred from general practitioners or hospitals were invited to Hammel Neurorehabilitation Centre and University Research Clinic by mail. A thorough assessment for eligibility was done by a neurologist and a psychiatrist and included:

1. A baseline questionnaire assessing the RPQ score, headache characteristics and symptom burden (see section regarding questionnaire data).
2. A neurological examination
3. A standardized psychiatric interview

Further details regarding the assessment procedure have been reported previously [18].

### Inclusion of controls

In 2022, we recruited a random cross-sectional sample of healthy, anonymous individuals aged 18–30 from the Blood Bank at Aarhus University Hospital, Denmark, matching the age group of the PPCS participants. The inclusion of healthy individuals allowed us to assess whether CGRP levels were altered in the PPCS participants at baseline, a crucial part of the primary outcome (outlined below). The healthy individuals did not participate in the RCT intervention, and the only demographic data available were age and sex. A sample size of 120 yielded a statistical power of 82% based on the effect size and standard deviations in a study in PTH [14].

### Serum samples

The blood samples were drawn from the antecubital vein using serum tubes (Vacutainer Cat 368815 or Cat 367896) and were allowed to clot for 30 min at room temperature. Subsequently, the blood samples were centrifuged for 10 min at 2880 g at 20 °C. The serum was then collected and initially frozen to  – 20 °C, and within 24 h moved to  – 80 °C until analysis. The samples were stored in 1.5 mL polypropylene tubes (Sarstedt, Nümbrecht, Germany). The preanalytical methodology was the same in samples from healthy individuals and PPCS participants, except the latter samples underwent one freeze–thaw cycle due to the measurement of other biomarkers for another study (not yet published, ClinicalTrials.gov NCT05812742). No protease inhibitors were added, and all participants were non-fasting.

### Primary outcome

The primary outcome was serum CGRP concentrations at baseline and follow-up. The baseline blood samples were obtained in connection with the assessment of eligibility 2–6 months after the concussion. The follow-up samples were obtained approximately 7 months later after the completion of the intervention/reference treatment. In contrast, the samples from the anonymous healthy individuals (blood donors) were obtained during their routine voluntary blood donations and no follow-up samples were collected.

### Questionnaire data/patient-reported outcomes

Headache data were retrieved from a previously published headache phenotype study conducted in the same PPCS participants [19]. In brief, the PPCS participants filled out a comprehensive headache questionnaire in connection with the assessment of eligibility. It contained data on monthly headache days, duration, current headache pain measured on the visual analogue scale (VAS), questionnaire data on the Headache Impact Test (HIT-6) [20], and allowed classification according to The International Classification of Headache Disorders 3rd edition [21]. Furthermore, questionnaire data on the RPQ, the Short Form (36) Health Survey (SF-36) [22], the Bodily Distress Syndrome Checklist (BDS) [23], Whiteley-8 (WI-8) [24], the “limiting behavior” and “all-or-nothing behavior” subscales from The Behavioural Responses to Illness Questionnaire (BRIQ) [25], The Brief Illness Perception Questionnaire (B-IPQ) [26], Symptom Checklist 8 AD (SCL-8AD) [27], and the Perceived Stress Scale (PSS) [28] were obtained from the RCT-study [15]. Supplementary Table 1 provides an overview of each questionnaire.

### CGRP assay

We analyzed the serum samples using a commercial enzyme-linked immunosorbent assay (ELISA) from Bertin Bioreagent which targeted α-CGRP and β-CGRP [29] (Cat No: A05481, Montigny le Bretonneux, France, batch no. 121 & 123). To reduce matrix effects, all calibration curves and quality controls (QCs) were prepared in CGRP-free serum. We prepared CGRP-free serum by incubating a pool of samples from healthy individuals and PPCS participants with CGRP antibodies (Cat No: A19482, Bertin Bioreagent, batch no. 0222) overnight at 4 °C on a tilt shaker. This was then filtered in accordance with Bertin’s instructions. Analysis of three replicates of the resulting CGRP-free serum, using the same methodology as for the study samples, revealed that the depletion was successful (< 2 pg/mL). To assess freeze–thaw stability, we spiked CGRP-depleted serum with 125 pg/mL using the provided CGRP standard in the kit, followed by freezing at  – 80 °C. The CGRP concentration remained stable averaging 141.3 pg/mL (SD: 25.4) for three freshly spiked replicates at 125 pg/mL, compared with 122.9 pg/mL (SD: 10.4) after one freeze–thaw-cycle. The observed difference was thus well below the < 20% deviation criterion specified in method validation guidelines [30].

Subsequently, the study samples were thawed at room temperature and analyzed using a calibration curve (7.8–1000 pg/mL) and quality control (125 pg/mL), both freshly prepared in CGRP-free serum. The assay procedure was performed following the manufacturer’s instructions. In short, after loading the samples, the tracer was added, and the plate was incubated overnight (16–20 h at 4 °C). Subsequently, Ellman’s reagent was added, and the resulting color intensity was analyzed with a microplate spectrophotometer (Synergy H1, BioTek, Winooski, The United States (USA)) at 410 nm after 75 min of incubation. Samples from patients and healthy individuals were included on each plate in an equal ratio to ensure representation of both groups. The position of the samples was varied between plates to reduce any bias from plate position. Analyses were conducted by the same experienced bioanalytical technician who was unblinded to the sample group (healthy or PPCS), but was blinded to sex and the hypothesis of the study. A calibration curve was included on each plate and plotted using Graphpad Prism (Graphpad Software, San Diego, USA) from which data were interpolated. The best fit was obtained using a second order polynomial regression (R^2^ ≥ 0.99, Suppl. Figure 1). CGRP values below limit of detection were replaced with the limit of detection reported by the manufacturer (2 pg/mL), and similarly, values exceeding the detection range were replaced with the highest value of the calibration curve (1000 pg/mL). Dilution of samples exceeding 1000 pg/mL was not performed due to insufficient sample material.

The mean QC concentration on all eight plates was 177.2 pg/mL (SD: 27.3) which was higher than the nominal concentration (125 pg/mL). However, the QCs were highly reproducible with an inter-assay coefficient of variation (CV) of 15.4% and an intra-assay CV ranging from 0% to 13% (median: 6.0%).

### Statistical analysis

Since the data were not normally distributed (Suppl. Figure 1), non-parametric tests were employed. CGRP concentration differences between PPCS participants and healthy individuals were assessed with the Wilcoxon rank-sum test, and adjustment for gender was done by stratified analyses. Changes at follow-up in PPCS participants were analyzed with the Wilcoxon signed-rank test. Non-parametric 95% confidence intervals (CI) were generated by bootstrapping the median CGRP difference, computing the resulting 2.5th and 97.5th percentiles across 10,000 replicates. For the primary outcomes, the significance level was set at p ≤ 0.050.

The effect of the non-pharmacological intervention on delta CGRP values (follow-up minus baseline) was evaluated by comparing the delta CGRP values in the treatment arms using the Wilcoxon rank-sum test. This was not included as a primary outcome, since we did not expect the intervention to alter CGRP levels, which was stated in the preregistered analysis plan.

In an exploratory analysis, we correlated serum CGRP levels with headache days, headache duration and current headache pain (VAS score) at baseline using Spearman’s rank correlation. Kruskal-Wallis test was used to test serum CGRP differences between headache phenotypes. Additionally, we assessed the correlation of CGRP levels to patient-reported outcomes (RPQ, HIT-6, SF-36, BDS, WI-8, BRIQ, B-IPQ, SCL-8AD, and PSS) using Spearman’s rank correlation. Finally, we assessed the correlation between delta CGRP and the delta value of the questionnaire data (follow-up minus baseline) using Spearman’s rank correlation. In total, 40 analyses were planned in the exploratory analyses, and the significance level was thus adjusted to p = 0.05/40 = 0.0013 (Bonferroni correction).

All statistical analyses were two-tailed and conducted using Stata 17 for Windows (StataCorp, College Station, USA), and graphs were created using Graphpad Prism.

## Results

**Fig. 1:**
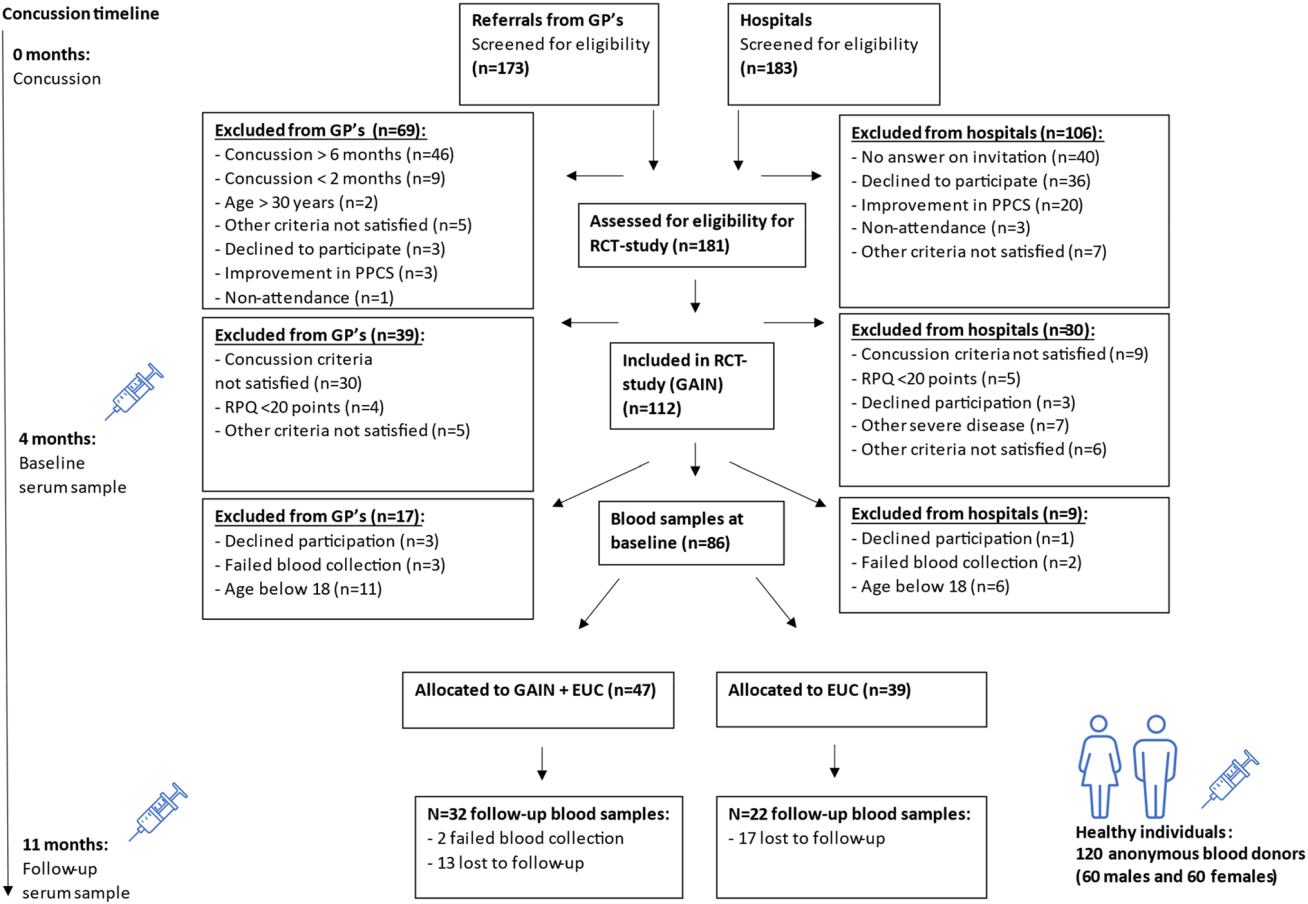
Flow-chart of inclusion procedure. The first blood sample was collected at a median of 4 months after the concussion at baseline of the RCT study before randomization. The second blood sample was obtained 7 months later, at approximately a median of 11 months after the trauma. For baseline comparisons, blood samples from a healthy cross-sectional group of blood donors were included, and these individuals did not participate in the intervention. *PPCS* Persistent post-concussion symptoms, *GP* General practitioner, *RCT* Randomized controlled trial, *GAIN* acronym for the intervention, Get going After concussIoN (novel non-pharmacological intervention), *EUC* Enhanced usual care (reference treatment)

Figure 1 depicts the inclusion procedure and shows that the majority (86 of 112) of the participants from the RCT provided blood samples at baseline at a median of 3.9 months after the trauma (IQR: 3.2–4.7). Most of the participants were female (78%), were on sick-leave (55%), and experienced a concussion in a traffic accident (31%) (Table 1). PTH was highly prevalent in the study as evidenced by 83 of 86 participants reporting headache. The typical participant had a constant headache (38%), 15–31 headache days a month (63%), and had a mixed phenotype between migraine-like and tension-type headache (41%), followed by migraine-like headache alone (34%) (Table 2). Follow-up blood samples were provided by 13 male and 41 female PPCS participants. Serum samples from healthy individuals were collected from 60 anonymous females with a mean age of 25.2 (SD: 2.8) and 60 anonymous males with a mean age of 26.0 (SD: 2.3).

**Table 1:** Characteristics of participants with PPCS four months post-trauma at baseline of the RCT

Demographic variables	PPCS female (n = 67)	PPCS male (n = 19)	Total PPCS (n = 86)
Age (mean, SD)	23.5 (3.3)	24.4 (4.6)	23.7 (3.6)
Months since trauma (median, IQR)	3.8 (3.1–4.7)	3.9 (3.2–5.0)	3.9 (3.2–4.7)
*Recruited from* (*n*, %)			
Emergency room	32 (47.8%)	7 (36.8%)	39 (45.3%)
General practitioner	35 (52.2%)	12 (63.2%)	47 (54.7%)
*Trauma mechanism* (*n*, %)			
Traffic accident	21 (31.3%)	6 (31.6%)	27 (31.4%)
Fall	13 (19.4%)	7 (36.8%)	20 (23.2%)
Hit by object	14 (20.9%)	2 (10.5%)	16 (18.6%)
Sports	16 (23.9%)	3 (15.8%)	19 (22.1%)
Assault	3 (4.5%)	1 (5.3%)	4 (4.7%)
*Job status* (*n*, %)			
Missing	2 (3.0%)	1 (5.3%)	3 (3.5%)
Full-time on sick leave	19 (28.4%)	7 (36.8%)	26 (30.2%)
Part-time on sick leave	19 (28.4%)	2 (10.5%)	21 (24.4%)
Full-time job/education	20 (29.9%)	8 (42.1%)	28 (32.6%)
Part-time job/education	3 (4.5%)	1 (5.3%)	4 (4.7%)
Other	4 (6.0%)	0	4 (4.7%)
*Education* (*n*, %)			
Missing	3 (4.5%)	2 (10.5%)	5 (5.8%)
Basic school (7–11 years)	13 (19.4%)	6 (31.6%)	19 (22.1%)
Upper secondary education (13–14 years)	33 (49.3%)	9 (47.4%)	42 (48.8%)
Further education	18 (26.9%)	2 (10.5%)	20 (23.3%)
*Pre-injury medical treatment* (*n*, %)			
Missing	4 (6.0%)	1 (5.3%)	5 (5.8%)
Mental disorder*	3 (4.5%)	0	3 (3.5%)
Somatic disorder**	7 (10.4%)	3 (15.8%)	10 (11.6%)
Use of oral contraceptives	16 (23.9%)	0	16 (18.6%)
*Pre-injury yearly headache days* (*n*, %)			
Missing	2 (3.0%)	0	2 (2.3%)
No headache	20 (29.9%)	6 (31.6%)	26 (30.2%)
1–7 days	15 (22.4%)	8 (42.1%)	23 (26.7%)
8–14 days	15 (22.4%)	1 (5.3%)	16 (18.6%)
15–30 days	8 (11.9%)	2 (10.5%)	10 (11.6%)
31–60 days	5 (7.5%)	1 (5.3%)	6 (7.0%)
61–90 days	2 (3.0%)	1 (5.3%)	3 (3.5%)

*PPCS* Persistent post-concussion symptoms, *RCT* Randomized controlled trial

*Depression/anxiety or attention deficit hyperactivity disorder (ADHD)

**Examples include medications for asthma, hypertension, hypothyroidism, acne, Crohn’s disease etc.

**Table 2:** Headache characteristics and patient-reported outcomes in participants with PPCS four months post-trauma at baseline of the RCT

Clinical data	PPCS female (n = 67)	PPCS male (n = 19)	Total PPCS (n = 86)
*Headache phenotype* (*n*, %)			
Missing	3 (4.5%)	0	3 (3.5%)
Migraine-like	25 (37.3%)	4 (21.1%)	29 (33.7%)
Tension-type-like	11 (16.4%)	4 (21.1%)	15 (17.4%)
Mixed (migraine-like + tension-type-like)	28 (41.8%)	7 (36.8%)	35 (40.7%)
Trigeminal autonomic cephalalgias-like (TACs)	0	2 (10.5%)	2 (2.3%)
Unclassified	0	2 (10.5%)	2 (2.3%)
*Headache days in last 4 weeks* (*n*, %)			
Missing	3 (4.5%)	1 (5.3%)	4 (4.7%)
1–2 days	0	1 (5.3%)	1 (1.2%)
3–7 days	5 (7.5%)	2 (10.5%)	7 (8.1%)
8–14 days	15 (22.4%)	3 (15.8%)	18 (20.9%)
15–31 days	43 (64.2%)	11 (57.9%)	54 (62.8%)
Uncertain	1 (1.5%)	1 (5.3%)	2 (2.3%)
*Duration of typical headache attack* (*n*, %)			
Missing	3 (4.5%)	0	3 (3.5%)
Few minutes – 15 min	2 (3%)	0	2 (2.3%)
15 min – 3 h	12 (17.9%)	4 (21.0%)	16 (18.6%)
4 h – 3 days	23 (34.3%)	4 (21.0%)	27 (31.4%)
Constant headache / No attacks	22 (32.8%)	11 (58.0%)	33 (38.3%)
Other/unspecified	5 (7.5%)	0	5 (5.8%)
*Current use of medication* (*n*, %)			
Missing	3 (4.5%)	0	3 (3.5%)
Paracetamol/Acetaminophen	54 (80.6%)	17 (89.5%)	71 (82.6%)
NSAIDs (e.g. ibuprofen)	33 (49.3%)	6 (31.6%)	39 (45.3%)
Aspirin	6 (9.0%)	4 (21.1%)	10 (11.6%)
Opioids (tramadol or morphine)	5 (7.5%)	3 (15.8%)	8 (9.3%)
Antidepressant medication (e.g. amitriptyline)	4 (6.0%)	0	4 (4.7%)
Migraine medication (e.g. triptans)	2 (3.0%)	1 (5.3%)	3 (3.5%)
*Additional PROs* (*Mean*, *SD*)			
HIT-6 (scale: 36–78)	65.0 (4.1)	62.7 (5.6)	64.5 (4.6)
RPQ (scale: 0–64)	38.3 (8.6)	35.4 (8.4)	37.6 (8.6)
Bodily pain domain on SF-36 (scale: 0–100)	35.2 (20.6)	37.3 (23.4)	35.7 (21.1)
BDS (scale: 0–100)	31.9 (12.2)	27.3 (13.2)	30.9 (12.5)

*PPCS* Persistent post-concussion symptoms, *PROs* Patient-reported outcomes, *HIT-6* Headache Impact Test, *RPQ* Rivermead Post-Concussion Symptoms Questionnaire, *SF-36* The Short Form (36) Health Survey, *BDS* Bodily Distress Syndrome Checklist, *RCT* Randomized controlled trial

### Primary/secondary outcomes

**Fig. 2:**
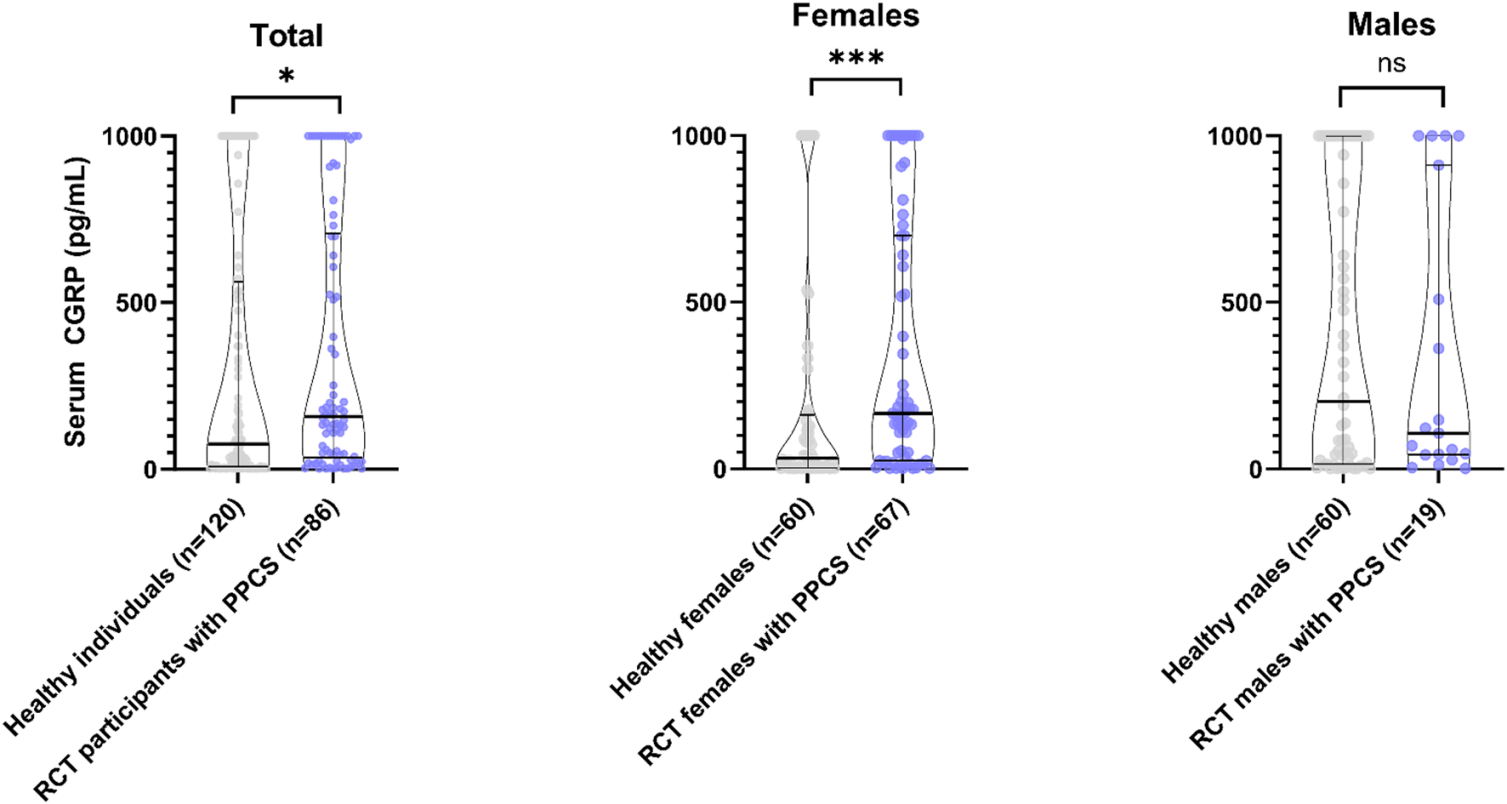
Violin plots of serum calcitonin gene-related peptide (CGRP) concentrations in baseline RCT participants with persistent post-concussion symptoms (PPCS) and healthy individuals not participating in the RCT. The median and interquartile range are displayed. Corresponding numbers are presented in Table 3. *p = 0.050. ***p = 0.0006. *CGRP* Calcitonin gene-related peptide, *RCT* Randomized controlled trial, *PPCS* Persistent post-concussion symptoms

**Fig. 3:**
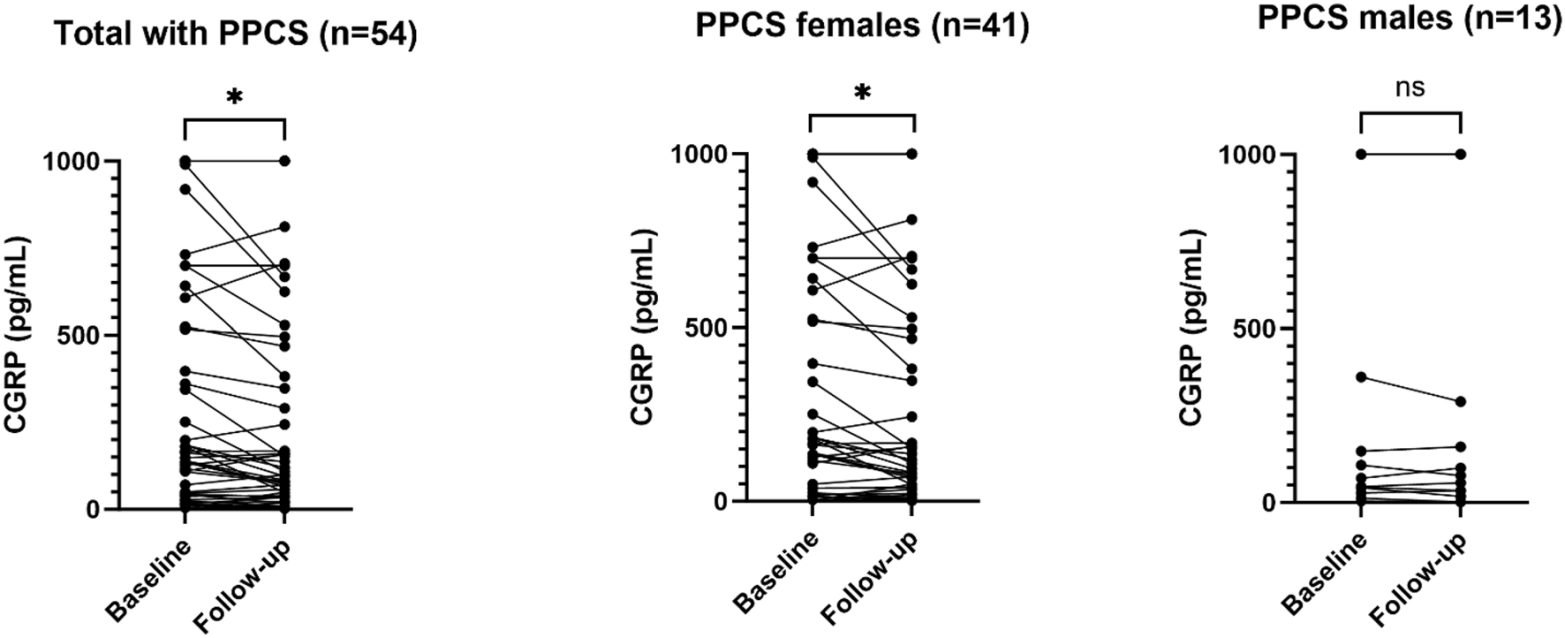
Baseline and follow-up serum calcitonin gene-related peptide concentrations in patients with persistent post-concussion symptoms. Baseline blood samples were taken at a median of 3.9 months after the trauma and the follow-up blood samples were taken at a median of 11.4 months after the trauma. Corresponding numbers are provided in Table 4. *p ≤ 0.05. *CGRP* Calcitonin gene-related peptide, *PPCS* Persistent post-concussion symptoms

Serum CGRP concentrations in PPCS participants at baseline of the RCT study and the healthy individuals are presented in Fig. 2 and Table 3. Median serum CGRP concentrations were doubled in PPCS participants compared to healthy individuals, with a median difference of 82.2 pg/mL (95% CI 17.7–145.6, p = 0.050). When stratifying for sex, it became evident that this difference was due to females with PPCS having a five times higher median concentration than healthy females (166.3 pg/mL vs. 32.1 pg/mL, p = 0.0006). No statistical difference was observed in males with PPCS (p = 0.83). Follow-up blood samples were collected at a median of 11.4 months (IQR: 10.3–12.7) after the trauma and following the completion of the non-pharmacological intervention/reference treatment. Figure 3 and Table 4 shows that CGRP levels decreased at follow-up in PPCS participants, with a median change of  – 1.3 pg/mL (95% CI  – 17.6–0, p = 0.024). The observed change was primarily attributed to females, who showed a median difference of  – 2.7 pg/mL (95% CI  – 37.4–0, p = 0.028) whereas males did not show any statistical difference (p = 0.54).

**Table 3:** Comparison of CGRP concentrations between baseline RCT participants with PPCS four months post-trauma and healthy individuals not partaking in the RCT

Sex (healthy vs. PPCS)	Healthy individuals		RCT participants with PPCS		PPCS vs. healthy	
	Mean CGRP (SD)	Median CGRP (IQR)	Mean CGRP (SD)	Median CGRP (IQR)	Median difference (95% CI)*	p^**^
Males (n = 60 vs. n = 19)	411.4 pg/mL (423.3)	201.8 pg/mL (16.5–1000)	340.6 pg/mL (413.8)	108.0 pg/mL (42.6–912.3)	– 93.8 pg/mL (– 437.9 to 371.3)	0.83
Females (n = 60 vs. n = 67)	198.0 pg/mL (337.7)	32.1 pg/mL (2.2–156.2)	350.7 pg/mL (376.9)	166.3 pg/mL (24.7–699.8)	134.2 pg/mL (75.2–190.2)	0.0006
Total (n = 120 vs. n = 86)	304.7 pg/mL (396.1)	76.3 pg/mL (7.9–554.0)	348.5 pg/mL (382.8)	158.5 pg/mL (37.9–699.8)	82.2 pg/mL (17.7–145.6)	0.050

*CGRP* Calcitonin gene-related peptide, *PPCS* Persistent post-concussion symptoms, *IQR* Interquartile range, *CI* Confidence interval

*Non-parametric 95% confidence intervals were obtained by taking the 2.5th and 97.5th percentiles of 10,000 bootstrapped replicates of the difference between the median in participants with PPCS and the median in healthy individuals

**Wilcoxon Rank-Sum Test

**Table 4:** Serum CGRP concentrations in PPCS participants with paired data

Sex	Median CGRP at baseline (IQR)	Median CGRP of paired differences (follow-up minus baseline)	p**
Males (n = 13)	46.6 pg/mL (28.0–148)	0 pg/mL IQR: – 10.5 to 6.6 95% CI* – 10.5 to 6.6	0.54
Females (n = 41)	166.3 pg/mL (24.7–607)	– 2.7 pg/mL IQR: – 64.8 to 2.0 95% CI* – 37.4 to 0	0.028
Total (n = 54)	138.6 pg/mL (24.7–524)	– 1.3 pg/mL IQR: – 55.3 to 3.0 95% CI* – 17.6 to 0	0.024

*PPCS* Persistent post-concussion symptoms, *IQR* Interquartile range, *CI* Confidence interval

*Non-parametric 95% confidence intervals were obtained by taking the 2.5th and 97.5th percentiles of 10,000 bootstrapped replicates of the median of the paired difference (follow-up minus baseline)

**Wilcoxon signed-rank test

There was no effect of the non-pharmacological intervention on CGRP levels: The median delta CGRP concentration was  – 3.5 pg/mL (IQR:  – 64.8–0) in the reference treatment (EUC), and 0 pg/mL (IQR:  – 46.4–14.9) in the intervention arm (GAIN), and there was no statistical difference (95% CI of median difference:  – 15.7–47.3, p = 0.33).

CGRP concentrations ranged from 2 pg/mL to 1000 pg/mL, indicating large interindividual variations in CGRP concentrations (Suppl. Figure 1). However, within each individual, baseline and follow-up concentrations showed a strong correlation (rho = 0.95, p < 0.0001).

### Exploratory analyses at baseline

**Fig. 4:**
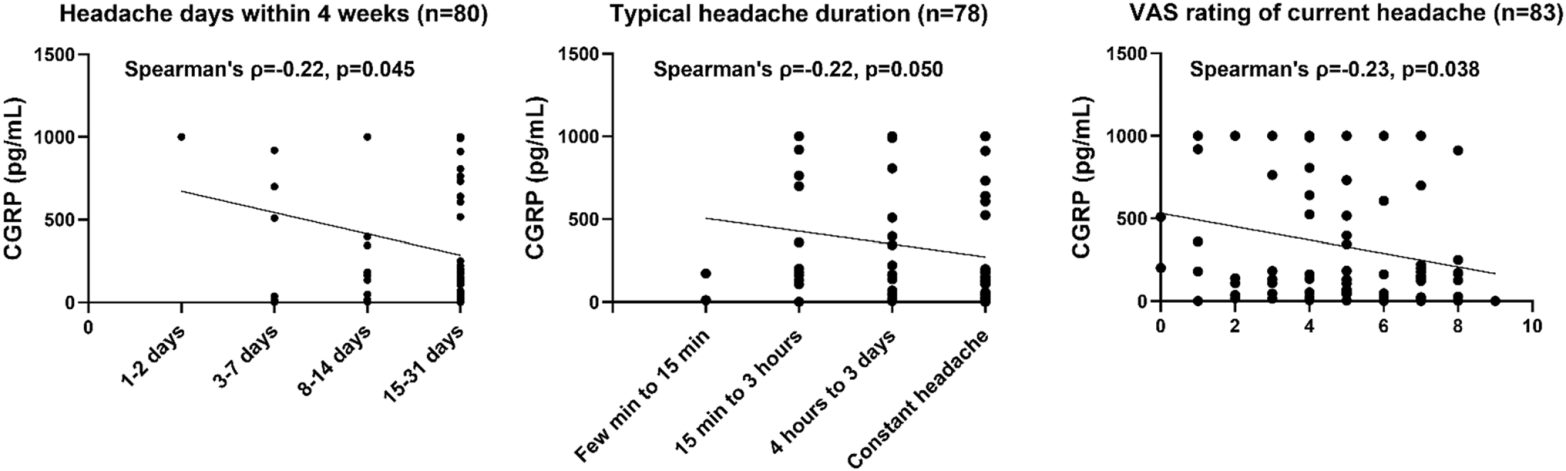
Correlation between serum calcitonin gene-related peptide and headache days, duration, and current headache pain at baseline. Significance level: 0.0013 (Bonferroni corrected). *CGRP* Calcitonin gene-related peptide, *VAS* Visual analog scale

**Fig. 5:**
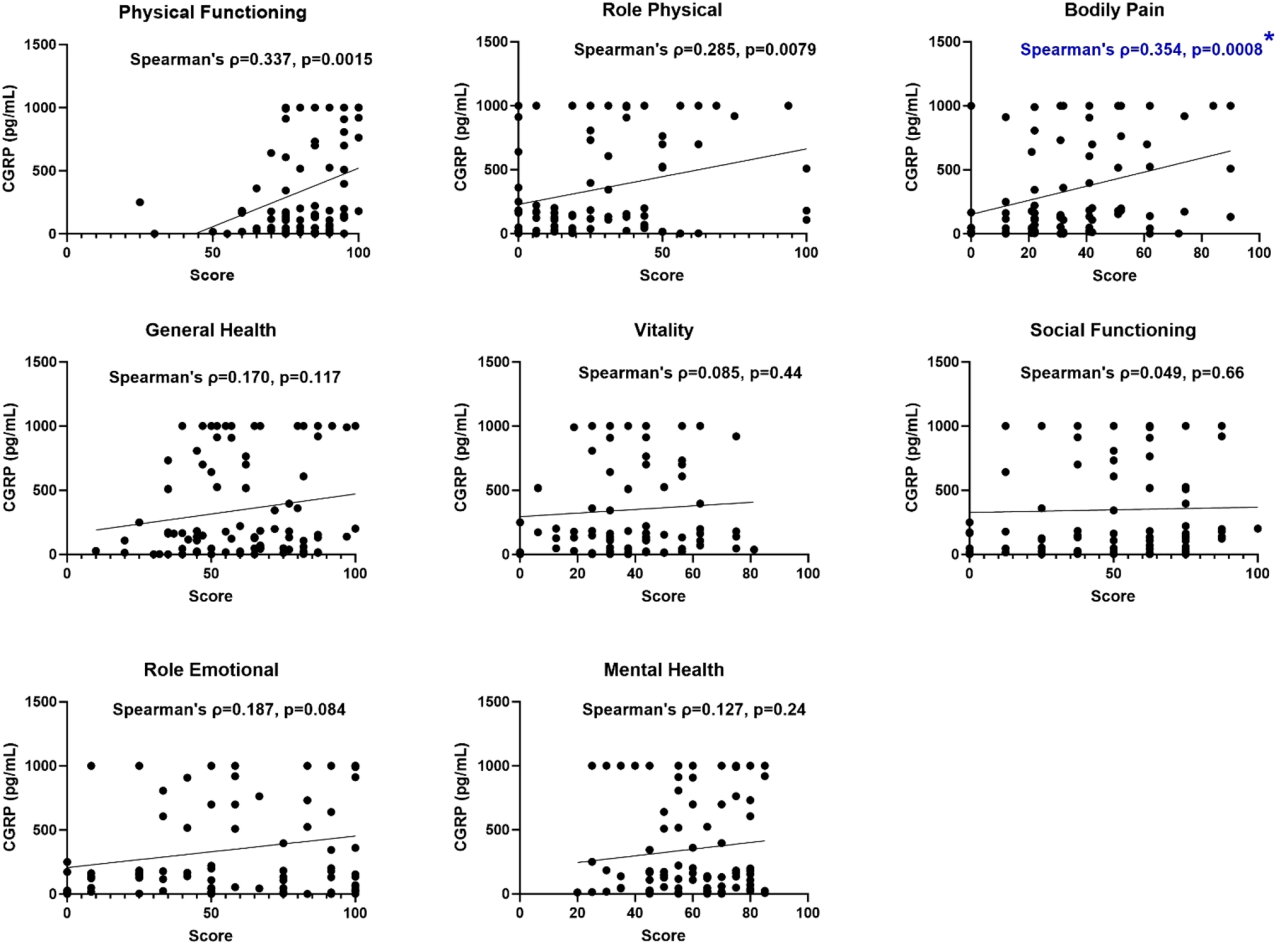
Explorative analysis of serum calcitonin gene-related peptide and The Short Form (36) Health Survey at baseline (n = 86). Higher score indicates *better* health. Significance level: 0.0013 (Bonferroni corrected). The blue color marks the analysis that survived the Bonferroni correction

**Fig. 6:**
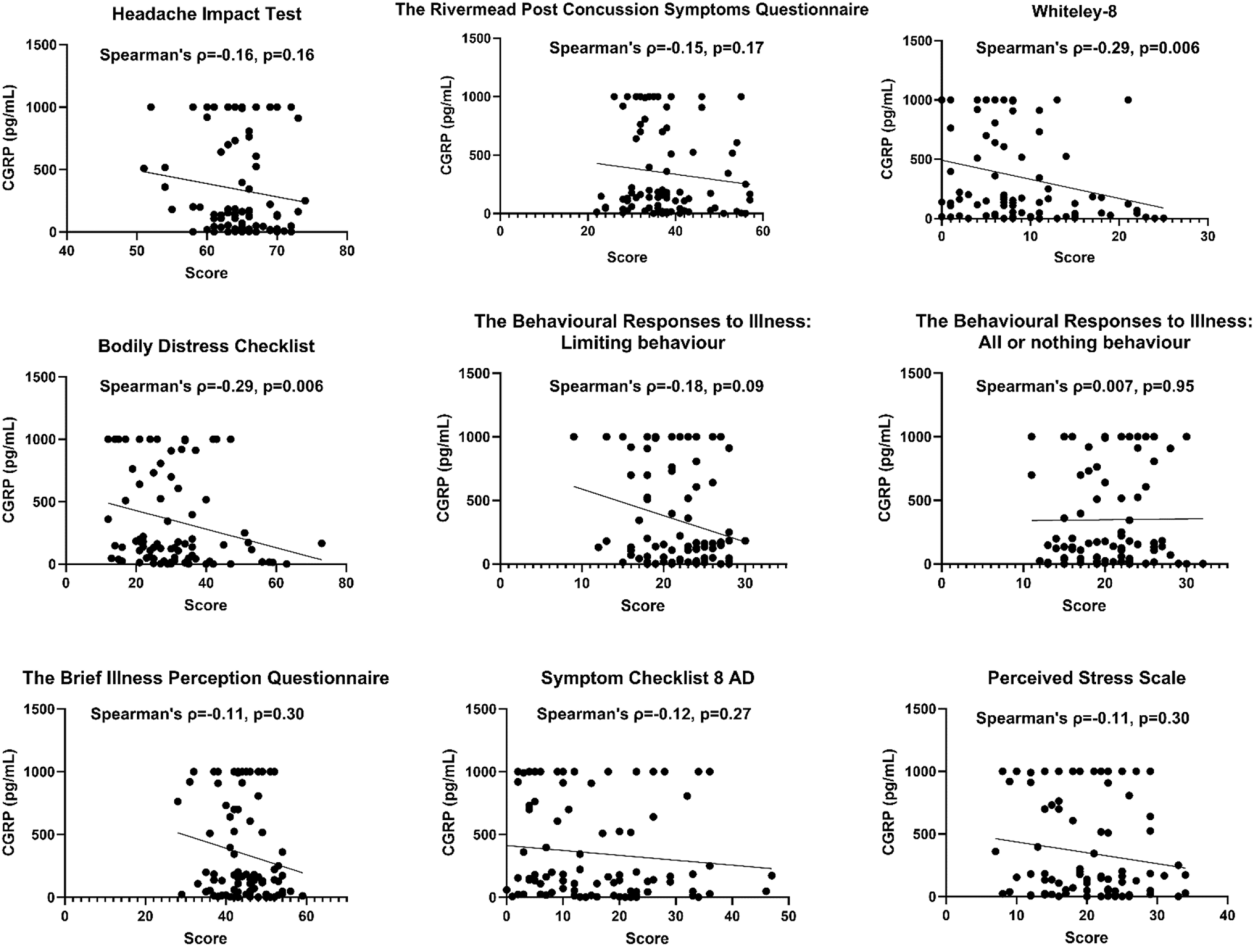
Explorative analysis of serum calcitonin gene-related peptide and patient-reported outcomes at baseline. Sample sizes was n = 83 for the Headache Impact Test, and n = 86 for the remaining questionnaires. Significance level: 0.0013 (Bonferroni corrected)

At baseline, high CGRP concentrations were associated with a more favorable outcome in several patient-reported outcome measures. For example, higher serum CGRP correlated weakly with fewer headache days, shorter headache duration, and less headache pain with p-values ranging from 0.038 to 0.050 at baseline (rho ≈  – 0.23) (Fig. 4). However, the Kruskal–Wallis test showed no CGRP concentration difference between headache phenotypes (p = 0.67). Further exploratory analyses (Figs. 5 and 6) showed that higher serum CGRP was correlated with a better physical function, physical health, and less bodily pain on the SF-36 questionnaire (rho = 0.29–0.35, p ≤ 0.0079). Likewise, higher CGRP levels were correlated with a smaller symptom burden as measured by the BDS and WI-8 questionnaires (rho =  – 0.29, p = 0.006). However, the only correlation that remained statistically significant after Bonferroni correction was the bodily pain domain on the SF-36 questionnaire (rho = 0.35, p = 0.0008).

An additional observation unrelated to PPCS was a higher median CGRP concentration among healthy males compared with healthy females as shown in Table 3 (201.8 pg/mL vs. 32.1 pg/mL, p = 0.0025), with a median difference of 169.7 pg/mL (95% CI: 14.2–496).

### Exploratory analyses at follow-up

**Fig. 7:**
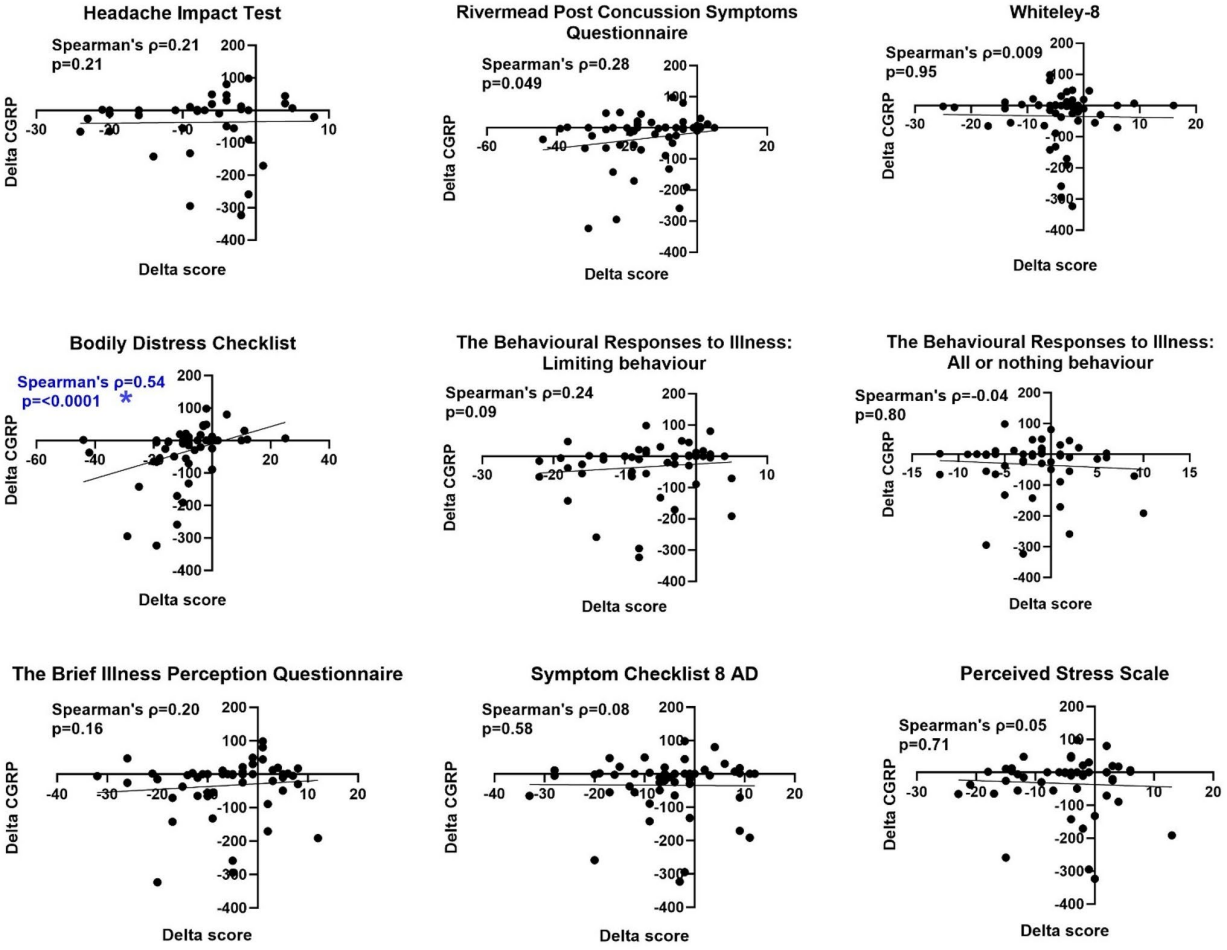
Change in CGRP (follow-up minus baseline) versus change in patient-reported outcomes (follow-up minus baseline). The sample size was n = 37 for the Headache Impact Test, and n = 50/51 for the remaining questionnaires. Significance level: 0.0013 (Bonferroni corrected). The blue color marks the analysis that survived the Bonferroni correction

The correlations between the change in CGRP concentrations (delta CGRP) and the change in questionnaire scores (delta score) from baseline to follow-up were examined (follow-up minus baseline). Unlike the baseline data, the follow-up questionnaire data and blood samples were obtained at different days, and the median time difference was 27 days (IQR: 9–50). Changes in headache days, duration, current headache pain, and changes in the SF-36 questionnaire scores were not correlated to changes in the CGRP concentration (Suppl. Figures 2 & 3). However, Fig. 7 shows that a reduction in CGRP at follow-up was associated with an improved RPQ and BDS-score, and the latter finding remained significant even after Bonferroni correction (rho = 0.54, p < 0.0001). A post hoc analysis revealed that the significant difference was mainly driven by an improvement in general symptoms such as headache and dizziness as well as cardiopulmonary/autonomic symptoms (Suppl. Table 2).

## Discussion

This study aimed to examine serum CGRP levels in participants with PPCS including PTH 2–6 months after a concussion. A novel finding is that elevated serum CGRP concentrations were observed in PPCS participants compared with healthy individuals. At follow-up, we observed a statistically significant reduction in CGRP levels, which correlated with a reduction in symptom levels. Although elevated CGRP levels have not previously been reported in PPCS, it is a common finding in migraine [31], and the clinical similarities suggest a potential role for CGRP in the pathophysiology of PTH. In fact, a recent study showed that CGRP infusion in patients with PTH could induce migraine-like headache [32]. In our study, > 70% reported headache with migraine-like features, suggesting the possible involvement of CGRP in this cohort as well. Interestingly, treatment with a CGRP antibody (erenumab) reduced the intensity and frequency of headache in a recent open-label study in PTH patients, suggesting its potential as a future treatment for PTH, similar to its use in migraine [33]. However, the evidence is limited, and placebo-controlled RCT studies are needed to draw firm conclusions.

The increased CGRP levels at baseline, and the reduction in CGRP levels at follow-up in PPCS participants were driven by females in this study. Interestingly, this is in accordance with a recent animal study showing increased serum CGRP concentrations after 7 days in female rats, but not in males after an induced head injury [34]. Females have a greater risk of developing PPCS/PTH [35], prompting speculation about a potential link between CGRP and this sex difference, similar to observations in migraine [36]. However, because of the limited sample size of males with PPCS in the present study (n = 19), firm conclusions on CGRP differences between PPCS males and healthy males are not possible due to a power issue, as evidenced by the wide confidence interval (Table 3).

Our findings of higher CGRP concentrations are in contrast with our primary hypothesis predicting lower CGRP concentrations in PPCS patients than those in healthy individuals. Our hypothesis was based on a previous study, which demonstrated lower CGRP plasma concentrations in PTH patients than in healthy controls [14]. The difference could be attributed to the inclusion criteria. In the present study, the mean disease duration was 4 months and only young participants were included compared to a mean disease duration of 49 months and a mean age of 36 in the previous study.

### Exploratory analyses – baseline data

Unexpectedly, we observed that higher CGRP levels correlated with fewer headache days, shorter headache duration and reduced current headache pain at baseline (rho ≈  – 0.23). Similarly, elevated CGRP levels correlated with a lower symptom burden, although bodily pain measured by the SF-36 was the only finding that remained statistically significant after Bonferroni correction. The fact that high CGRP concentrations are correlated with a more positive outcome, contradicts findings in migraine [31] and the majority of pain-related studies [37]. However, it could align with the findings in the previously mentioned PTH study [14]. This earlier study included patients refereed to a specialized headache clinic, indicating more severe headaches. Interestingly, their study demonstrated lower CGRP levels in PTH than controls and the same tendency toward an inverse relationship with monthly headache days although it was not statistically significant (rho =  – 0.11, p = 0.27). This tendency was shown in a recent study conducted in post-deployment soldiers as well (rho =  – 0.12, p = 0.063) [38]. It is thus possible that more severe PTH are associated with lower CGRP levels. An explanation for lower CGRP given in the previous PTH study was that constant headache may result in the depletion of CGRP in trigeminal afferents [14]. This speculation was based on the finding that CGRP tissue levels were depleted in rats following capsaicin injection in the paw skin and sciatic nerve [39]. Whether capsaicin-mediated pain is comparable to the pain in headache is unknown.

However, other explanations can account for our findings as well. CGRP is a neuropeptide with several physiological functions unrelated to headache [13]. In the aforementioned animal study, CGRP inhibition with antibodies in concussed female rats did not alleviate cephalic pain hypersensitivity, raising questions about the role of peripheral CGRP in headache in females [34]. In contrast, there are several studies showing that CGRP inhibition could alleviate symptoms in rodents with migraine-like behavior in both males and females [40]. Furthermore, in a recent study on severe traumatic brain injury in humans, high CGRP concentrations were correlated with a lower risk of mortality, indicating a potential beneficial role for CGRP [41]. Additionally, an animal study showed that CGRP may have a favorable impact on peripheral nerve regeneration [42]. Further studies are needed to establish the role of CGRP in concussion and PTH.

A noteworthy observation, unrelated to PPCS, in our study was that healthy males had higher serum levels of CGRP than healthy females. This align with a recent study in post-deployment soldiers [38], but contrasts with a previous study that showed the opposite in females, particularly among those using oral contraceptives [43]. Studies investigating sex differences in CGRP among healthy individuals are limited, and it was not the primary aim of this study; further research is needed to draw definite conclusions.

### Exploratory analyses – follow-up data

At follow-up we showed that a reduction in CGRP levels was correlated with improved symptom levels measured by the BDS questionnaire. The post hoc analysis revealed that the reduction in CGRP correlated especially with an improvement in headache, dizziness and autonomic/cardiopulmonary symptoms (Suppl. Table 2). This suggests that a CGRP reduction could be linked to a positive physiological response. A major limitation in the follow-up data was that the questionnaire data and blood samples were collected at different time points, with a median time difference of 27 days. This could have contributed to the lack of an association between delta CGRP and change in headache days, duration, and pain, particularly when considering the limited response rate in the follow-up headache questionnaire (Suppl. Figure 2). Finally, the RCT intervention did not show any effect on CGRP concentrations at follow-up. This was expected and stated in the preregistered analysis plan since the RPQ-difference (symptom levels) was only 7 points between the intervention arms [15].

In conclusion of our exploratory analyses at baseline and follow-up, we found an association between CGRP levels and patient-reported outcomes measured by questionnaires, which is a rare finding. Future studies should replicate these findings, and additional research is needed to establish the role of CGRP in PPCS/PTH and concussion.

### CGRP assay

The CGRP concentrations in this study varied substantially with concentration levels ranging below detectable levels (< 2 pg/mL) to above 1000 pg/mL (Suppl. Figure 1). This wide concentration range is larger than those observed in migraine studies [31]. Apart from differences in inclusion criteria and demographic characteristics (such as sex), variations in assays and methodology can affect CGRP concentrations [44]. It is worth noting that a previous study using a similar methodology and same ELISA kit as our study showed a comparable concentration range, mean, and data distribution in healthy individuals (Suppl. Figure 4) [45].

### Strengths and limitations

This study had several strengths. The cohort was well-characterized, the concussion diagnosis was validated using the WHO criteria, and we had a relatively large sample size.

We used an ELISA-kit with no cross-reactivity with calcitonin, a peptide fragment of CGRP (CGRP position 8-37), amylin, and substance P according to the manufacturer. Furthermore, the assay was based on two antibodies for CGRP (sandwich ELISA) indicating its specificity for CGRP. In contrast, most other commercial ELISA kits have not investigated cross-reactivity and are only based on one antibody. Moreover, the QC’s and the calibration curves were constructed in study representative serum matrix, which should minimize the risk of matrix effects. Finally, we checked freeze–thaw stability of CGRP in serum to ensure that the reported concentrations in PPCS participants were accurate.

This study also had limitations. The QC’s systematically produced higher than nominal concentrations (170 pg/mL vs. 125 pg/mL), which indicates a slight overestimation of the concentrations in general. However, more importantly, the QC’s were reproducible with an inter-assay and intra-assay CV ≤ 15%. Furthermore, although the manufacturer reported no cross-reactivity between similar molecules, it cannot be ruled out that cross reactivity exists with a related peptide biomarker. However, this seems unlikely since CGRP was completely depleted in CGRP-free serum which was a pool of samples from both patients and healthy individuals. Since samples from patients and healthy individuals were evenly distributed on each ELISA plate, we do not expect these assay related factors to affect the conclusion of this study.

Preanalytical stability of CGRP is another point of concern. Prolonged storage and lack of protease inhibitors may decrease CGRP concentrations [45], although there is conflicting evidence on this matter [46]. In our study, patient samples underwent one freeze–thaw cycle and had a longer storage duration (up to 8 years), in contrast to the samples from healthy individuals (1 year of storage). Despite these possible limitations, our study still revealed significantly higher CGRP levels in PPCS patients than in healthy individuals. A further limitation of the study was the presence of multiple symptoms in addition to headache among PPCS participants, a consequence of the inclusion criteria requiring an RPQ score of 20 or higher. This diversity of symptoms prevented a clear identification of the exact causes of increased CGRP levels in PPCS participants.

Finally, the healthy individuals consisted of anonymous blood donors, for whom no demographic data were available. Danish blood donors tend to have a higher self-reported health and healthier lifestyle than non-donors, which may introduce a selection bias known as “the healthy donor effect” [47]. Although the mechanisms are poorly understood, lifestyle-related factors, such as higher weight, blood pressure [48], and exercise [49], might increase CGRP-concentrations. Since we lacked data on lifestyle factors in both groups, it cannot be ruled out that the observed differences might be partly explained by variations in lifestyle factors rather than PPCS/PTH. The potential confounding effects of preexisting migraines must also be considered since the headache questionnaire utilized did not allow accurate classification of headaches prior to the concussion [19]. However, 75% of the PPCS participants reported less than 15 headache days a year pre-trauma, and none reported using migraine medications before the concussion (Table 1). Furthermore, since migraine is generally not a contraindication for becoming a blood donor, the group of healthy individuals may also include individuals with migraine, thereby reducing the likelihood that this condition skews our findings. Regardless of potential confounding variables, the observed fivefold increase in median CGRP concentration in females with PPCS, compared with healthy individuals, is substantial. For this difference to be solely attributed to confounding factors, these factors would have to exert a strong influence on CGRP levels.

In conclusion, our data are strongly suggesting a role for CGRP in PTH. Future studies should aim to independently verify whether this is the case, preferably in a population with blood samples available before the head trauma (which can be done in athletes), to clearly establish a causal link between CGRP and concussion/PTH in humans. Furthermore, future studies should investigate whether CGRP targeted therapies are effective in PTH in a placebo-controlled RCT design.

### Supplementary Information

Below is the link to the electronic supplementary material.

Supplementary file1 (PDF 236 KB)

Supplementary file2 (PDF 468 KB)

## Data Availability

Anonymized raw data are not publicly available, but are available for research upon request.
