# Supplementary material for: Serum calcitonin gene-related peptide in patients with persistent post-concussion symptoms, including headache: a cohort study
Source: J Neurol. 2024 Jan 17;271(5):2458–72. doi: 10.1007/s00415-024-12181-y (PMC11055722; doi:10.1007/s00415-024-12181-y)
Supplement: Supplementary file 1 — Supplementary file1 (PDF 236 KB) [file 415_2024_12181_MOESM1_ESM.pdf]

Supplementary Tables

Suppl. Table 1: Overview of each questionnaire in the explorative analyses

Questionnaire	Abbreviation	Description	Rating system	Score range
The Rivermead Post-Concussion Questionnaire	RPQ	A 16-item questionnaire designed to measure the severity of post-concussion symptoms.	A list of post-concussion symptoms (e.g. headache) is rated from a 0 (not experienced) to 4 (a severe problem).	0-64
Headache Impact Test	HIT-6	A six-item questionnaire designed to measure the burden of headaches with items concerning pain, social functioning, role functioning, vitality, cognitive functioning, and psychological distress	Each item is rated on a 5-point scale (6=never, 8= rarely, 10= sometimes, 11= very often, 13= always).	36-78
The Short Form (36) Health Survey	SF-36	A 36-item questionnaire designed to measure health status. It is subdivided into eight dimensions: Physical functioning, role limitations due to physical health, bodily pain, general health, vitality, social functioning, role limitations due to emotional problems, and mental health	In six of the eight dimensions, the responses are rated on three or six-point scales, and the remaining two (role limitations) are answered by yes/no questions. Each dimension is converted to a scale from 0 (worst health) to 100 (best health)	0-100
Bodily Distress Syndrome Checklist	BDS	A 25-item questionnaire designed to measure the most common somatic symptoms experienced in functional disorders. It is subdivided into cardiopulmonary/autonomic symptoms, gastrointestinal symptoms, musculoskeletal symptoms, and general symptoms (e.g. headache).	Each symptom is rated from a scale from 0 (not bothered at all) to 4 (bothered a lot) within a four week time frame	0-100
Whiteley-8	WI-8	An eight-item questionnaire designed to measure health anxiety.	Each item is rated from a scale from 0 “not bothered at all” to 4 “a lot” within a four week time frame	0-32

Corresponding author:
Peter Preben Eggertsen
Hammel Neurorehabilitation Centre and University Research Clinic, Denmark
Email: peter.eggertsen@gmail.com

The Behavioral Responses to Illness Questionnaire (BRIQ)	BRIQ	A questionnaire designed to measure the behavior during the acute phase of an illness.<br>Two subscales from BRIQ were used in this study: All-or-nothing behavior (7 items) and limiting behavior (6 items)	Each item is rated from a scale from 1 “Not at all” to 5 “Every day”	All or nothing behavior: 7-35<br><br>Limiting behavior: 6-30
The Brief Illness Perception Questionnaire	B-IPQ	A nine-item questionnaire designed to measure illness perception. Item 5 was removed (symptom burden) since this is measured by several other questionnaires. Item 9 was removed since it does not contain a score.	Each item is rated from 0 (no affect at all) to 10 (severely affects my life)	0-70
Symptom Checklist 8 AD	SCL-8AD	A 13-items questionnaire measuring mental distress, including anxiety, depression and emotional distress	Each item is rated from 0 “not bothered at all” to 4 “a lot” within a 4 week time frame	0-52
Perceived Stress Scale (PSS)	PSS-10	A 10-item questionnaire designed to measure the perception of stress	Each item concerns the frequency of certain feelings and thoughts within the last month, and is rated from a scale from 0 (Never) to 4 (Very often). The sum score is achieved by reverse scoring the positive items, and then summing all items.	0-40

Corresponding author:
Peter Preben Eggertsen
Hammel Neurorehabilitation Centre and University Research Clinic, Denmark
Email: peter.eggertsen@gmail.com

Suppl. Table 2: Post hoc analysis
The delta scores of the subitems of the Bodily Distress Syndrome Checklist (BDS) were correlated to the delta calcitonin gene-related peptide (CGRP) levels in participants with persistent post-concussion symptoms (follow-up minus baseline, n=51). The participants assessed the severity of 25 symptoms over the past four weeks on a scale of 0 (not at all), 1 (a bit), 2 (somewhat), 3 (quite a bit), 4 (a lot).

**During the last four weeks, have you been bothered by….**	Baseline BDS score (median, IQR)	Median delta BDS score (follow-up minus baseline, IQR)	Correlation of delta BDS score to delta CGRP (Spearman's rho)	P-value of correlation
Cardiopulmonary/autonomic symptoms				
Palpitations/heart pounding?	0 (0-1)	0 (-1-0)	0.36	0.0091
Precordial discomfort?	0 (0-1)	0 (-1-0)	0.16	0.27
Breathlessness without exertion?	0 (0-2)	0 (-1-0)	0.50	0.0002
Hyperventilation?	0 (0-0)	0 (0-0)	0.16	0.27
Hot or cold sweats?	1 (0-2)	0 (-2-0)	0.27	0.056
Dry mouth?	0 (0-1)	0 (-1-0)	0.38	0.0065
Gastrointestinal symptoms				
Frequent loose bowel movements?	0 (0-2)	0 (0-0)	0.013	0.93
Abdominal pain?	1 (0-2)	0 (-1-1)	0.06	0.67
Feeling bloated/full of gas/distended?	1 (0-2)	0 (-1-1)	0.10	0.49
Diarrhea?	0 (0-0)	0 (0-0)	0.08	0.59
Regurgitations?	0 (0-1)	0 (-1-0)	0.05	0.71
Nausea?	1 (0-3)	-1 (-1-0)	0.20	0.15
Burning sensation of the upper part of stomach/epigastrium?	0 (0-1)	0 (0-0)	-0.01	0.97
Musculoskeletal symptoms				
Pain in arms or legs?	0 (0-1)	0 (-1-0)	0.06	0.66
Muscular aches or pain?	1 (0-3)	0 (-1-0)	0.17	0.25
Pain in the joints?	0 (0-2)	0 (-1-0)	0.02	0.88
Feeling paresis or localized weakness?	0 (0-1)	0 (0-0)	0.22	0.13
Back ache?	1 (0-2)	0 (-1-0)	0.09	0.55
Pain moving from one place to another?	0 (0-0)	0 (0-0)	-0.04	0.78
Unpleasant numbness or tingling sensations?	0 (0-1)	0 (0-0)	0.20	0.16
General symptoms				
Concentration difficulties?	3 (3-4)	-1 (-2-0)	0.10	0.51
Excessive fatigue?	3 (3-4)	0 (-2-0)	0.27	0.053
Headache?	3 (3-4)	-1 (-2-0)	0.37	0.0069
Impairment of memory?	3 (2-4)	0 (-1-0)	0.35	0.011
Dizziness?	2 (1-3)	-1 (-2-0)	0.47	0.0005
Total BDS score	29 (21-37)	-8 (-15 to -2)	0.54	<0.0001

Corresponding author:
Peter Preben Eggertsen
Hammel Neurorehabilitation Centre and University Research Clinic, Denmark
Email: peter.eggertsen@gmail.com
