# Supplementary material for: Serum calcitonin gene-related peptide in patients with persistent post-concussion symptoms, including headache: a cohort study
Source: J Neurol. 2024 Jan 17;271(5):2458–72. doi: 10.1007/s00415-024-12181-y (PMC11055722; doi:10.1007/s00415-024-12181-y)
Supplement: Supplementary file 2 — Supplementary file2 (PDF 468 KB) [file 415_2024_12181_MOESM2_ESM.pdf]

Title: Serum Calcitonin Gene-Related Peptide in Patients with Persistent Post-Concussion Symptoms, Including Headache: A Cohort Study
Journal of Neurology
Authors: Peter Preben Eggertsen, Johan Palmfeldt, Henrik Winther Schytz, Debbie Hay, Rikke Katrine Jentoft Olsen, Jørgen Feldbæk Nielsen

Supplementary Figures

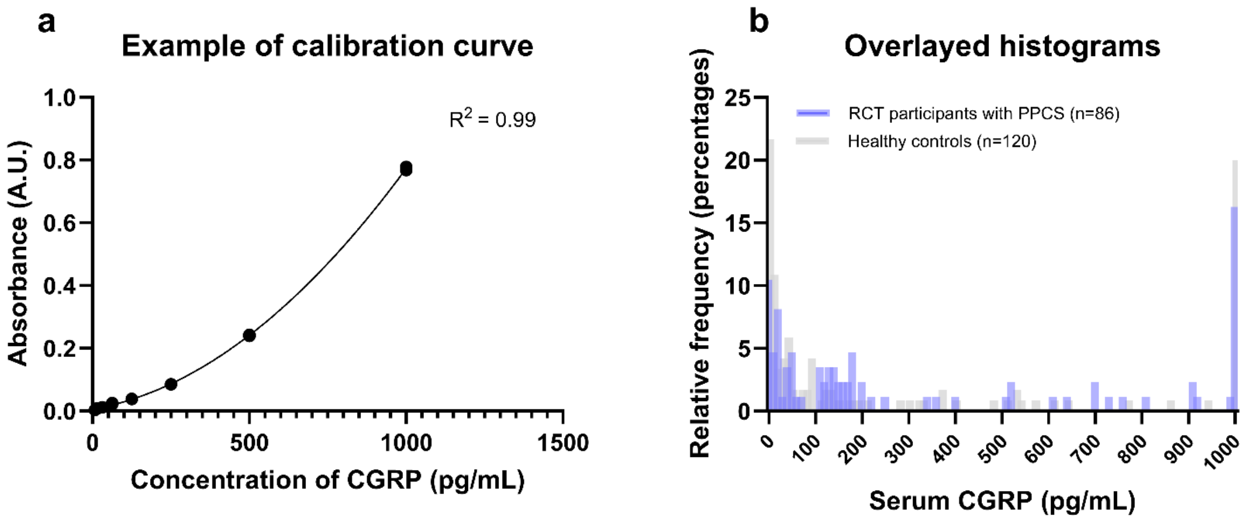

Supplementary Figure 1: Example of calibration curve and histogram of CGRP concentrations
(a) Example of a calibration curve used to interpolate values in the study samples (second order polynomial regression). The model fit was excellent with an $R^2 = 0.99$, and the mean accuracy of the back-calculated concentrations for each calibrator level (7.8 pg/mL – 1000 pg/mL) ranged from 99% to 112%. (b) Histogram showing the distribution of serum CGRP concentrations in healthy individuals and baseline RCT participants with PPCS. As can be seen, the data in both groups do not follow a normal distribution. In total, 64/206 samples (31%) were outside the detection limits of the assay. More specifically, 26 (12.6%) of the samples were at or below the detection limit of the assay (≤2 pg/mL), and similarly, 38 samples (18.4%) were at or above the detection limit (≥1000 pg/mL). The distribution is similar to what has been observed in a previous study using the same assay (Suppl. Figure 4).
Abbreviations: RCT: Randomized controlled trial, PPCS: Persistent post-concussion symptoms, A.U.: Arbitrary Units

Corresponding author:
Peter Preben Eggertsen
Hammel Neurorehabilitation Centre and University Research Clinic, Denmark
Email: peter.eggertsen@gmail.com

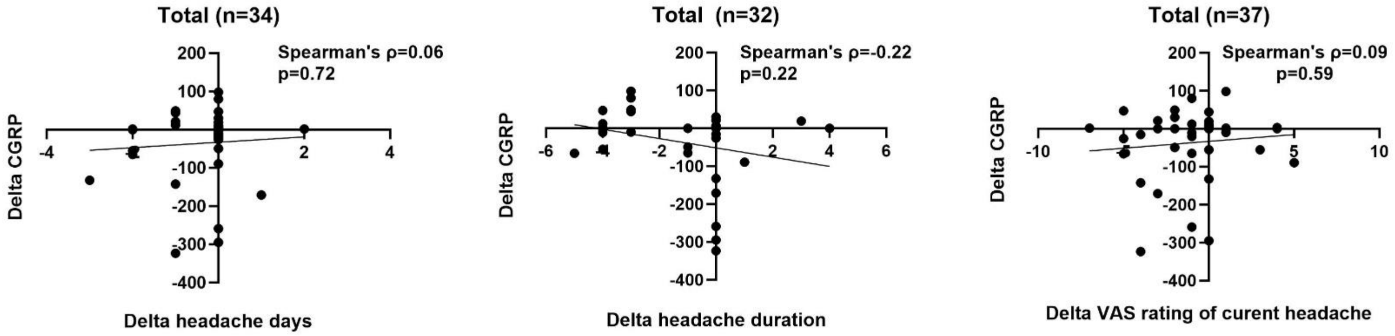

Supplementary Figure 2: Change in CGRP (Follow-up minus Baseline) versus Change in Headache Characteristics (Follow-up minus Baseline).
Headache days and headache duration were categorical variables. Therefore, the delta unit is arbitrary.
Significance level: 0.0013 (Bonferroni corrected)

Corresponding author:
Peter Preben Eggertsen
Hammel Neurorehabilitation Centre and University Research Clinic, Denmark
Email: peter.eggertsen@gmail.com

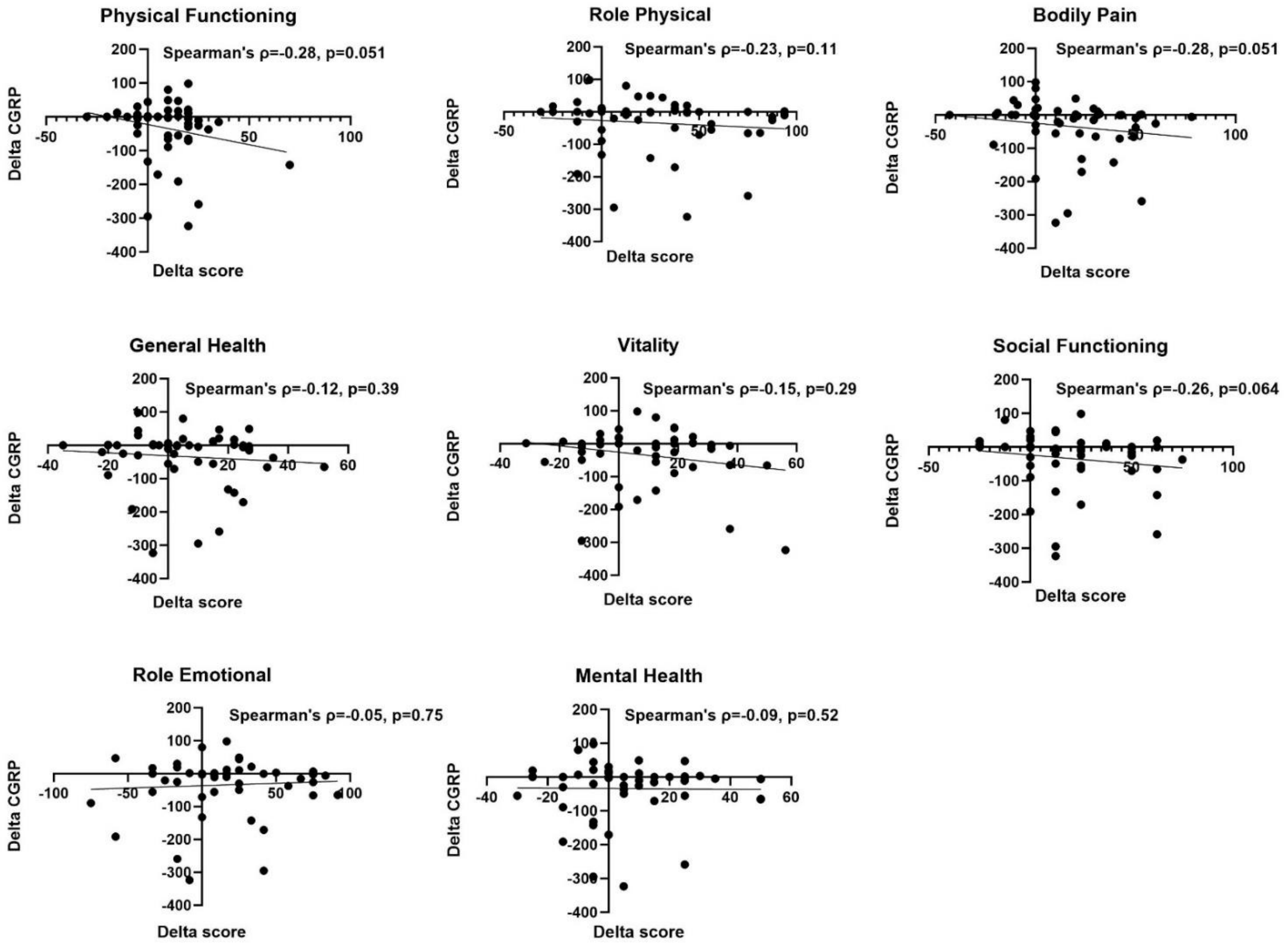

Supplementary Figure 3: Change in CGRP (Follow-up minus Baseline) versus Change in SF36 Questionnaire Data (Follow-up minus Baseline).
N=50, significance level: 0.0013 (Bonferroni corrected)

Corresponding author:
Peter Preben Eggertsen
Hammel Neurorehabilitation Centre and University Research Clinic, Denmark
Email: peter.eggertsen@gmail.com

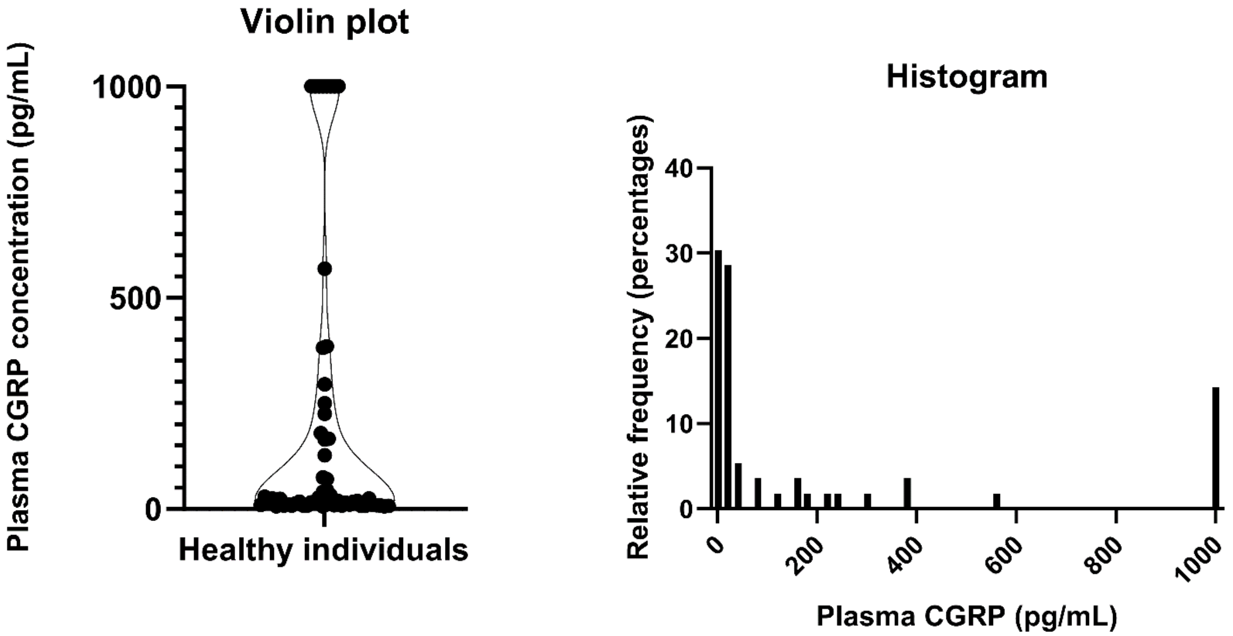

Supplementary Figure 4: Violin Plot and Histogram of Calcitonin Gene-Related Peptide Concentrations in a Previous Study
Plasma calcitonin gene-related peptide concentrations (CGRP) concentrations in 56 healthy individuals were extracted from a previously published study using the same assay and methodology (Table 5 in Messlinger et. al, ref no. 45). Data >1000 pg/mL were replaced with 1000 to align with the present study. As can be seen, the data distribution is similar to the distributions observed in the present study (Figure 2 & Suppl. Figure 1).

Corresponding author:
Peter Preben Eggertsen
Hammel Neurorehabilitation Centre and University Research Clinic, Denmark
Email: peter.eggertsen@gmail.com
